# Dynamic response of deepwater test string under fluctuations in axial force and internal pressure

**DOI:** 10.1038/s41598-023-38901-4

**Published:** 2023-07-20

**Authors:** Qiaolei Sun, Yuwei Liu, Long Deng, Jiangang Wang, Ding Feng

**Affiliations:** 1 School of Mechanical Engineering, Yangtze University, Jingzhou, 434023 Hubei China; 2 Hubei Engineering Research Center for Oil & Gas Drilling and Completion Tools, Jingzhou, 434023 Hubei China

**Keywords:** Engineering, Fossil fuels

## Abstract

In this study, a mechanical model suitable for deepwater test string was proposed. An analysis of the dynamic response of the test string under different frequencies, different water depths and different fluctuation amplitudes was carried out by using the finite element method based on the change in the internal pressure and axial force measured. The results of the analysis showed that the response parameters (maximum stress and maximum deformation) tended to be stable after one period of fluctuation in the axial force and half a period of fluctuation in the internal pressure, respectively. When a sine waveform fluctuation in the internal pressure and axial force occurred, the response parameters increased with an increase in the amplitude of the fluctuation and increased with an increase in the frequency of fluctuation, and the amplitude of variation decreased with an increase in the fluctuation period. Under fluctuation in the axial force, the response parameter decreased with an increase in the water depth. The response parameter decreased first and then increased with an increase in the water depth when the fluctuation in the internal pressure occurred with a sine waveform. The maximum deformation and stress of the test string always changed with a change in the load when the fluctuation in the internal pressure and axial force had a sine waveform, and the test string under a load with a sine waveform was prone to periodic fatigue failure. The relevant conclusions provide a basis for the analysis and prevention of fatigue failure in test strings.

## Introduction

With the exploration and development of the deepwater gas field in the South China Sea, the demand for and quantity of deepwater test operations have increased to evaluate the production capacity of the related exploratory wells^1,2^. In the course of deepwater oil and gas well testing, the axial force and internal pressure of the test string may change greatly through the influence of drift, swing, heave and other factors such as floating movement and changes in the internal output of the test string and the action of the well opening and closing caused by the load of the deepwater environment^3–5^. Under the complicated stress conditions of the test string system itself, the safety of the test string will be affected to a certain extent. In order to ensure the safety of deepwater test operations, it is necessary to carry out an analysis of the dynamic response of deepwater test strings under fluctuations in the load.

In the relevant research on the existing test operations, Korolev^6^, Zenith^7^ and Bottomley et al.^8^ presented different approaches to reduce the cost of testing oil or gas wells. Andok et al.^9^ proposed a series of multi-test events for testing wells. Xing^10^, Shahbazi^11^ and Boukadi et al.^12^ proposed different analysis models for testing wells. Park et al.^13^classified well testing methods by their characteristics. The abovementioned research mainly focused on the test methods and the methods used for analysis and evaluation of the tests. Some mechanical analyses of test strings have mainly focused on land operations^14,15^. For deepwater testing, this is only equivalent to a study of the test string under the mud line.

For a test string in the seawater section above the mud line, the current research has mainly included the influence of temperature on the telescopic change in the test string^16^, optimization of the time at all stages of the test process^17^, optimization of the process of deepwater test operations^18^, studies on testing the internal gas used as the critical carrying fluid^19^, analyses of the thread connection strength of the string between the test strings^20^, etc. In the area of dynamic responses, the research has mainly focused on the flow pipeline. Argueelles and Casanova^21^ presented a numerical approach to calculate the amplitudes of the steady-state response of a piping system, which considered dry friction between the pipes. Mathan and Prasad^22^ used FE to analyze the dynamic response of a piping system with gasketed flanged joints, and discussed the important parameters affecting the vibration. Mohammad et al.^23^ presented a simplified mathematical model of a pipe filled with liquid or gas subjected to dynamic loading, and developed asymptotic analytical and numerical methods for the analysis of the governing equation. Yan et al.^24^ presented an analytical model for the dynamic response of pipe-on-pipe impact, and used the explicit finite element code MSC/DYTRAN in numerical simulations. Zan et al.^25^ proposed a coupled time-domain numerical model to analyze the dynamic reactions of a deepwater S-laying vessel.

From the above mentioned research, we know that the existing studies on test operations and pipelines’ dynamic responses have mainly focused on the improvement of the test methods, the establishment of a mathematical model of predictive tests, generation of the pipeline’s pulsating pressure, the process of test strings, the safety of the string connections and so on. There is no system that can establish a mechanical model of the seawater section in a deepwater test string, and no dynamic analysis of a test string has been carried out under fluctuations in the axial force and internal pressure. Therefore, in this study, an analysis of the dynamic response of the test string under fluctuations in the internal pressure was carried out for a single short string (a single tube with a length of 10 m) of the seawater section of a deepwater test string.

The main purpose of this study was to establish a mechanical model of a deepwater test string, which mainly included calculation of the stress of the seawater section of the string, calculation of the axial force, and establishment of the model equation of transverse vibration and longitudinal vibration in the test string. On this basis, combined with the change in the axial force and internal pressure of a well in the field, an analysis of the dynamic response of the test string under different water depths, different amplitudes of fluctuation and different fluctuation periods was carried out by establishing the FE model of a single short string. The dynamic response of the axial forces under different forms of fluctuation was analyzed, and the law of the influence of different fluctuation parameters on the maximum stress and deformation of the string was obtained.

## Analysis of a mechanical model of a deepwater test string

### Introduction to the test string’s structure

**Figure 1:**
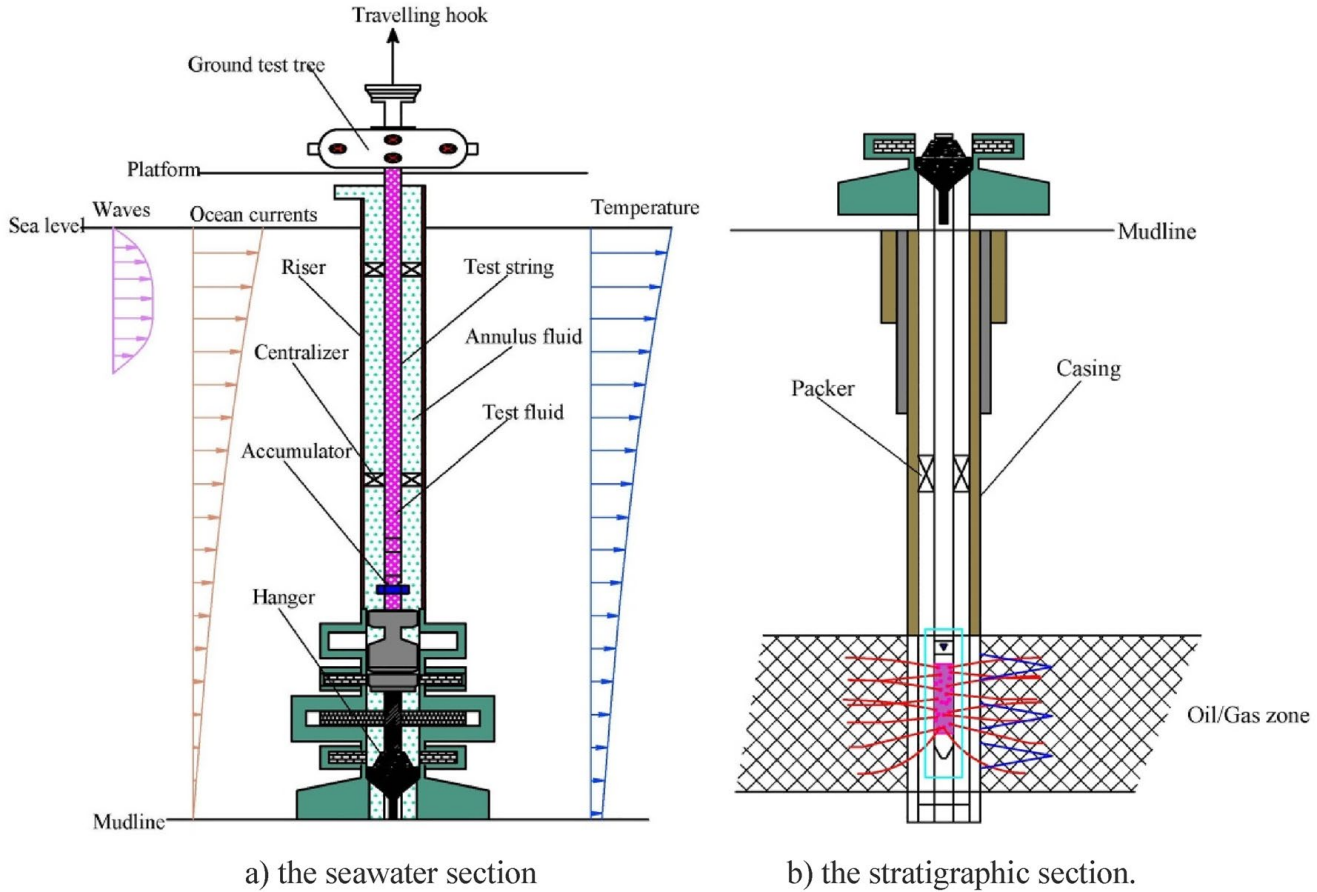
The structure of a deepwater test string.

As shown in Fig. 1, because of the widespread high temperature and high pressure of deepwater oil and gas well production layers, the related theoretical research, basic structure and principles that apply under the mud line are similar to those of terrestrial test strings. Therefore, the test string under the mud line can be studied by means of the research results of the mechanics related to land-based oil and gas well strings. The test string above the mud line (i.e., the seawater section) is located in the riser, the outside of which is subjected to annulus fluid, and the fluid thus produced acts upon the interior. At the same time, in the context of marine environmental loads, the riser and the drilling ship or platform will produce a certain amount of floating movement. Therefore, it is easy to produce large fluctuations in the axial load of the shaft upward of the test string, so the output needs to be adjusted according to the test’s requirements, and the fluctuation in the internal pressure of the test string is also obvious during the process of adjustment.

### A model for calculating the axial force of the seawater section of the string

**Figure 2:**
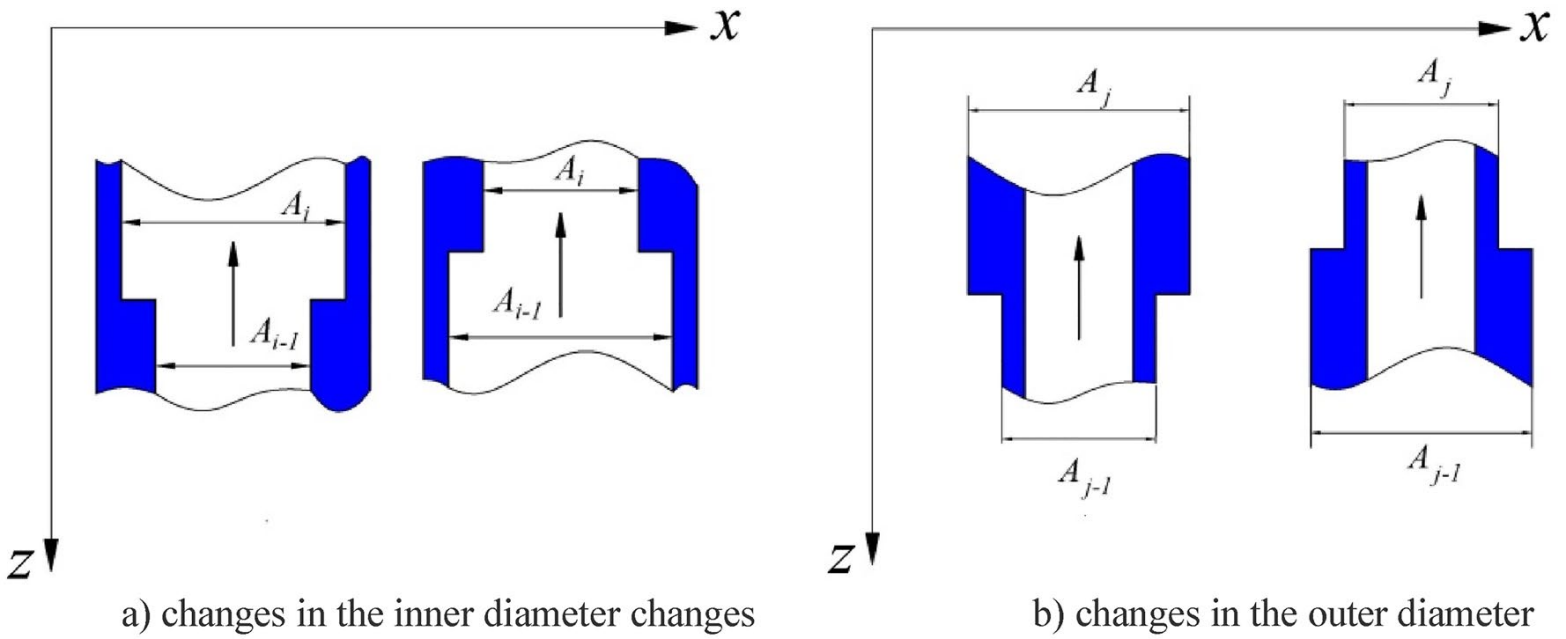
Changes in the inner and outer diameter of a test string.

For calculation of the axial force of the string under the action of internal and external pressure, extensive discussions have been carried out on the frictional force, the hydraulic force and the elongation and compression of the string^26–28^. In previous research, the authors increased the influence of the section with a variable diameter on the axial force in view of the structural characteristics of the test string. Since the test string in the seawater section is composed of multiple oil strings and functional components, there are many variable internal and external diameters, as shown in Fig. 2. At the same time, the internal pressure of the string changes greatly with different working conditions (going down, switching wells, perforation, etc.) during the test, so it was necessary to take changes in $F_{{\text{v}}}$ into account in the analysis of the test string.

Combined with the change in the pressure and the cross-sectional area of the variable inner and outer diameters of the test string, the test string $F_{{\text{v}}}$ can be expressed as

1 $$F_{\text{v}} = \sum\limits_{1}^{n} {p_{{{\text{o}}i}} } \Delta A_{i} \, \, + \, \, \sum\limits_{1}^{k} {p_{{{\text{o}}j}} } \Delta A_{j} \, \, \, \, \, \, \, \, \, \, \, \, i = 1,2,3 \ldots n\, \, \, \, \, \, \, j = 1,2,3 \ldots k\, \, \,$$

where $n$ represents the number of variable diameters for the outer diameter; $p_{{{\text{o}}i}}$ represents the pressure at the ith reduction in the inner diameter; $\Delta A_{i} \,$ represents the area of pressure acting on the variable inner diameter, where $\Delta A_{i} = A_{i} - A_{i - 1}$; $k$ is the number of variable diameters for outer diameter;$p_{{{\text{o}}j}}$ is the pressure at the jth reduction in the outer diameter; $\Delta A_{j} \,$ is the area of pressure acting on the variable outer diameter, $\Delta A_{j} = - A_{j} + A_{i - 1}$.

The axial force $F_{z} \left( {z,t} \right)$ of the seawater string can be expressed as

2 $$F_{z} \left( {z,t} \right) = F_{{\text{o}}} \left( t \right) - \int_{0}^{z} {\left[ {q_{{\text{m}}} \left( {z,t} \right) - \left( {\rho_{\text{fo}} A_{\text{ro}} - \rho_{{{\text{fi}}}} A_{{{\text{ri}}}} } \right)g - F_{{\text{f}}} \left( {z,t} \right)} \right]} {\text{d}}z + \frac{{EA_{{\text{s}}} }}{2z}\int_{0}^{z} {\frac{{{\text{d}}^{{2}} x}}{{{\text{d}}z^{2} }}} {\text{d}}z - F_{{\text{v}}} - T_{0} - F_{{\text{N}}}$$

where $F_{z} \left( {z,t} \right)$ is the true axial force of the test string at time t and depth z, in N; $F_{0} \left( t \right)$ represents the instantaneous tension of the test string at time t, in N; $q_{m}$ is the effective weight of the test string, in N; $\left( {\rho_{{{\text{fo}}}} A_{{{\text{ro}}}} - \rho_{{{\text{fi}}}} A_{{{\text{ri}}}} } \right)g$ is the imaginary force generated by the inner and outer fluids of the string, in N; $\rho_{{{\text{fo}}}}$ and $\rho_{{{\text{fi}}}}$ are the density of the outer annulus test fluid and the fluid in the string, respectively, in kg/m^3^; $A_{{{\text{ro}}}}$ and $A_{{{\text{ri}}}}$ are the outer cross-sectional area and inner cross-sectional area of the string, respectively, in m^2^; $F_{{\text{f}}} \left( {z,t} \right)$ is the frictional resistance between the inner/outer walls and the inner/outer fluid of the string, in N; $\frac{{EA_{{\text{s}}} }}{2z}\int_{0}^{z} {\frac{{{\text{d}}^{{2}} x}}{{{\text{d}}z^{2} }}} {\text{d}}z$ is the additional axial force generated by the transverse bending of the column, in N; $E$ is the elastic modulus of the string;$A_{{\text{s}}}$ is the effective sectional area of the string, in m^2^, $T_{0}$ is the true gravity of the string below the hanger, in N; ad $F_{{\text{N}}}$ is the support force at the hanger, in N.

The value of $q_{m}$ can be calculated by the formula deduced in the previous derivation^29^ as follows.

3 $$q_{{\text{m}}} = q_{{\text{s}}} k_{{\text{f}}}$$

where $q_{{\text{s}}}$ is the line weight of the string in the air, in N/m;$k_{{\text{f}}}$ represents the buoyancy coefficient, $k_{{\text{f}}} = 1 - \rho_{{{\text{fo}}}} /\rho_{{\text{s}}}$; and $\rho_{{\text{s}}}$ is the string’s density, in kg/m^3^.

### Vibration model of the string

The vibration model of the test string mainly includes longitudinal vibration and transverse vibration, in which the longitudinal vibration of the string under the inflow movement is combined with the characteristics of the completed string and the classical four equations of a pipe proposed by Skalak et al.^30^, assuming that the test string is a vertical section and considering the friction between the inner and outer fluids and the inner and outer walls of the string^31–33^. The equation can be expressed as follows.

4 $$\begin{gathered} \frac{\partial P}{{\partial z}} + \rho_{\text{fi}} \frac{\partial V}{{\partial t}} - g + \frac{{f_{\text{i}} \left( {V - \dot{u}_{z} } \right)\left| {V - \dot{u}_{z} } \right|}}{4R} = 0 \hfill \\ \frac{\partial V}{{\partial z}} + \left( {\frac{1}{K} + \frac{2R}{{Ee}}} \right)\frac{\partial P}{{\partial t}} - \frac{2\nu }{E}\frac{{\partial \sigma_{z} }}{\partial t} = 0 \hfill \\ \frac{{\partial \dot{u}_{z} }}{\partial t} - \frac{1}{{\rho_{{\text{s}}} }}\frac{{\partial \sigma_{z} }}{\partial t} - g - \frac{{\rho_{\text{fi}} f_{\text{i}} \left( {V - \dot{u}_{z} } \right)\left| {V - \dot{u}_{z} } \right|}}{{8\rho_{{\text{s}}} e}} + \frac{{\rho_{{{\text{fo}}}} f_{{\text{o}}} \dot{u}_{z} \left| {\dot{u}_{z} } \right|}}{{8\rho_{{\text{s}}} e}} = 0 \hfill \\ \frac{{\partial \dot{u}_{z} }}{\partial z} - \frac{1}{E}\frac{{\partial \sigma_{z} }}{\partial t} + \frac{\nu R}{{Ee}}\frac{\partial P}{{\partial t}} = 0 \hfill \\ \end{gathered}$$

where $P$ is the pressure of the fluid in the string, $V$ is the velocity of the fluid in the string, $\dot{u}_{z}$ is the speed of the movement of the string along the axial direction, $f_{{\text{i}}}$ is the friction coefficient between the fluid and the wall in the string, $K$ is the bulk modulus of the fluid, *R* is the string of the inner diameter, *e* is the thickness of the string’s wall, $\nu$ is the Poisson's ratio of the string,$f_{{\text{o}}}$ is the coefficient of friction between the annulus test fluid and the outer wall of the string, and $\sigma_{z}$ is the axial stress of the string.

Equation (4) is used to calculate the longitudinal vibration of the test string and comprehensively considers the Poisson coupling and friction coupling between the fluid inside and outside the test string and the string, which can explain better the equation of motion of the internal fluid during the longitudinal vibration of the test string, the continuity equation, the axial motion equation of the string and the relationship between the stress and the velocity of the string.

For the transverse vibration of the seawater test string, according to the existing methods of analysis^14,33^ without considering the coupling of the internal fluid and the string, the mathematical model of the transverse vibration of the string can be expressed as follows

5 $$\frac{{\partial^{2} }}{{\partial z^{2} }}\left( {EI\frac{{\partial^{2} x}}{{\partial z^{2} }}} \right) - \frac{{\partial^{{}} }}{\partial z}\left( {F_{z} \left( {z,t} \right)\frac{\partial x}{{\partial z}}} \right) - \frac{{q_{m} }}{g}\frac{{\partial^{2} x}}{{\partial z^{2} }} = F_{x} \left( {z,t} \right)$$

where $EI$ is the bending stiffness of the section of the test string, in N m^2^; $T\left( z \right)$ is the distribution of the axial force along the depth of string, in N; $q_{m}$ is the floating weight of the test string per unit of length, in N/m; and $F_{x} \left( {z,t} \right)$ is the distribution of the transverse force along the depth of the string, considering the actions of the internal and external fluid and the riser on the string.

It can be seen from Eqs. (4) and (5) that changes in the axial force, and the internal and external pressure of the seawater section of the test string have an obvious influence on the axial stress, transverse vibration and longitudinal vibration of the test string, and the change in axial stress can cause changes in the axial velocity and internal pressure of the string.

### Von Mises strength criterion

For the test operation, because of the high pressure and high temperature, the internal pressure will produce a certain fluctuation depending on the output and the different working conditions. Because the overall length of a test string is generally hundreds of meters or even several kilometers, it can be simplified to an equal-section cylinder, according to the Lame formula^34^, and the radial stress and hoop stress of the test string element can be expressed as follows.

6 $$\begin{gathered} \sigma_{{r}} = - \frac{{{{r_{{\text{o}}}^{2} } \mathord{\left/ {\vphantom {{r_{{\text{o}}}^{2} } {r^{2} }}} \right. \kern-0pt} {r^{2} }} - 1}}{{{{r_{{\text{o}}}^{2} } \mathord{\left/ {\vphantom {{r_{{\text{o}}}^{2} } {r_{i}^{2} }}} \right. \kern-0pt} {r_{\text{i}}^{2} }} - 1}}P - \frac{{1 - {{r_{{\text{i}}}^{2} } \mathord{\left/ {\vphantom {{r_{{\text{i}}}^{2} } {r^{2} }}} \right. \kern-0pt} {r^{2} }}}}{{1 - {{r_{{\text{i}}}^{2} } \mathord{\left/ {\vphantom {{r_{{\text{i}}}^{2} } {r_{{\text{o}}}^{2} }}} \right. \kern-0pt} {r_{{\text{o}}}^{2} }}}}q \hfill \\ \sigma_{\phi } = \frac{{{{r_{{\text{o}}}^{2} } \mathord{\left/ {\vphantom {{r_{{\text{o}}}^{2} } {r^{2} }}} \right. \kern-0pt} {r^{2} }} + 1}}{{{{r_{{\text{o}}}^{2} } \mathord{\left/ {\vphantom {{r_{{\text{o}}}^{2} } {r_{{\text{i}}}^{2} }}} \right. \kern-0pt} {r_{{\text{i}}}^{2} }} - 1}}P - \frac{{1 + {{r_{{\text{i}}}^{2} } \mathord{\left/ {\vphantom {{r_{{\text{i}}}^{2} } {r^{2} }}} \right. \kern-0pt} {r^{2} }}}}{{1 - {{r_{{\text{i}}}^{2} } \mathord{\left/ {\vphantom {{r_{{\text{i}}}^{2} } {r_{{\text{o}}}^{2} }}} \right. \kern-0pt} {r_{{\text{o}}}^{2} }}}}q \hfill \\ \end{gathered}$$

where $\sigma_{{r}}$ is the radial stress, $\sigma_{\phi }$ is the hoop stress, $r_{{\text{o}}}$ is external radius of the string, $r_{{\text{i}}}$ is the internal radius of the string, $r$ is the radius of string element and $q$ is the pressure of the annulus fluid.

According to the fourth strength theory, the equivalent stress $\sigma_{s}$ of the string can be expressed as follows.

7 $$\sigma_{s} = \sqrt {\frac{1}{2}\left[ {\left( {\sigma_{z} - \sigma_{r} } \right)^{2} + \left( {\sigma_{z} - \sigma_{\phi } } \right)^{2} + \left( {\sigma_{r} - \sigma_{\phi } } \right)^{2} } \right]}$$

During testing, when the heat exchange between the internal and external environment and the annulus fluid is not considered, the pressure of the annulus fluid can be calculated as follows

8 $$p_{{\text{o}}} = \rho_{{{\text{fo}}}} gz + P_{{0}}$$

where $P_{0}$ is the annulus pressure in the wellhead.

For the seawater section of the test string, because of the action of the upper drilling platform, the deep compensating device, the telescopic device, etc., the upper end will have a certain up and down motion, and the lower end may be regarded as having a small rotation caused by restrictions such as the hanger and the accumulator. During testing, the axial force and internal pressure of the test string must change during the different working conditions.

Since $\sigma_{z}$, $\sigma_{r }$ and $\sigma_{\phi }$ are related to the axial force, the internal pressure and the external pressure, fluctuations in the axial force and internal pressure will inevitably lead to a change in the stress and deformation of the string under conditions of constant external pressure. The influence of force and internal pressure on the dynamic response of the string was analyzed in by combining the actual test data of a well in the South China Sea and FE modeling. The transient dynamics analytical module of the FE software package Workbench was used to analyze different changes in the dynamic analysis, and this method has been applied in related studies and compared with other scholars' data^35,36^. In this study, the dynamic responses of the maximum stress and deformation of a single short string were obtained.The establishment of the theoretical model provides a basis for the calculation of axial force of test string, two-dimensional Fluid–structure interaction analysis, transverse vibration analysis and foundation strength calculation. And which also laid the foundation for the subsequent simulation results analysis of the paper.

## Establishment and loading of the FE models

### Modeling and material properties

**Figure 3:**
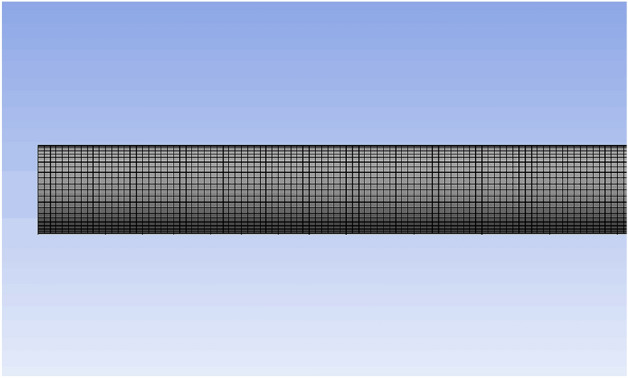
Grid diagram of a partial string.

According to the string parameters used during deepwater testing in the South China Sea, a single short string (a 10 m tube) with an equal section was selected for analyzing the dynamic response. The outer diameter of the string was 114.3 mm and the inner diameter was 85.85 mm. The FE model was modeling by Workbench directly, the model’s grid was Solid186, the number of grids was 108,102 and the number of nodes was 583,586. The grid diagram of the partial string is shown in Fig. 3.

The material model adopted the ideal elastoplastic model, an elastic modulus of *E* = 2.06 × 10^5^ MPa and Poisson's ratio $\nu$ = 0.3. The material selected for the string was 42CrMo, and the yield strength was $\sigma_{{\text{s}}}$ = 930 MPa.

### Model of the loading form and load size

According to the internal and external pressure of the string calculated in the previous work by our research group, the loading form was hydrostatic pressure, the variation was in accordance with the water depth, where the external pressure changed to 0.01274 MPa/m along the z-axis (water depth), and the internal pressure varied along the z-axis (water depth) to 0.0038 MPa/m. The reference datum could be set with the water depth.

**Figure 4:**
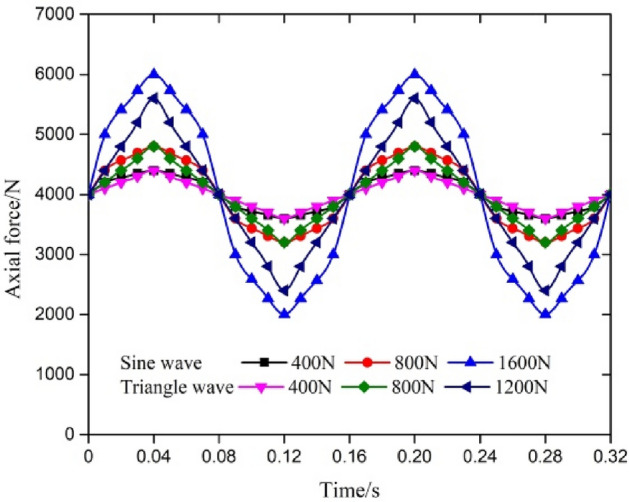
Transient variation in the axial force.

For the axial force loading, it can be seen from Eq. (2) that it is affected by many factors. The axial force has approximately the wave form of a triangular wave and a sine wave for a certain period of time. Based on the measured frequency of 3.125 Hz, the cycling time (0.16 s, 0.32 s, 0.48 s, 0.64 s and 0.8 s), different water depths (sea level, 325 m, 475 m and 660 m), sine waves and triangular waves with different amplitudes of axial force (400 N, 800 N and 1600 N), triangular waves were selected. In an analysis of two cycles, each cycle's load input point was 27 and each calculation step contained 5 substeps. The partial loading form of the transient loading value is shown in Fig. 4.

**Figure 5:**
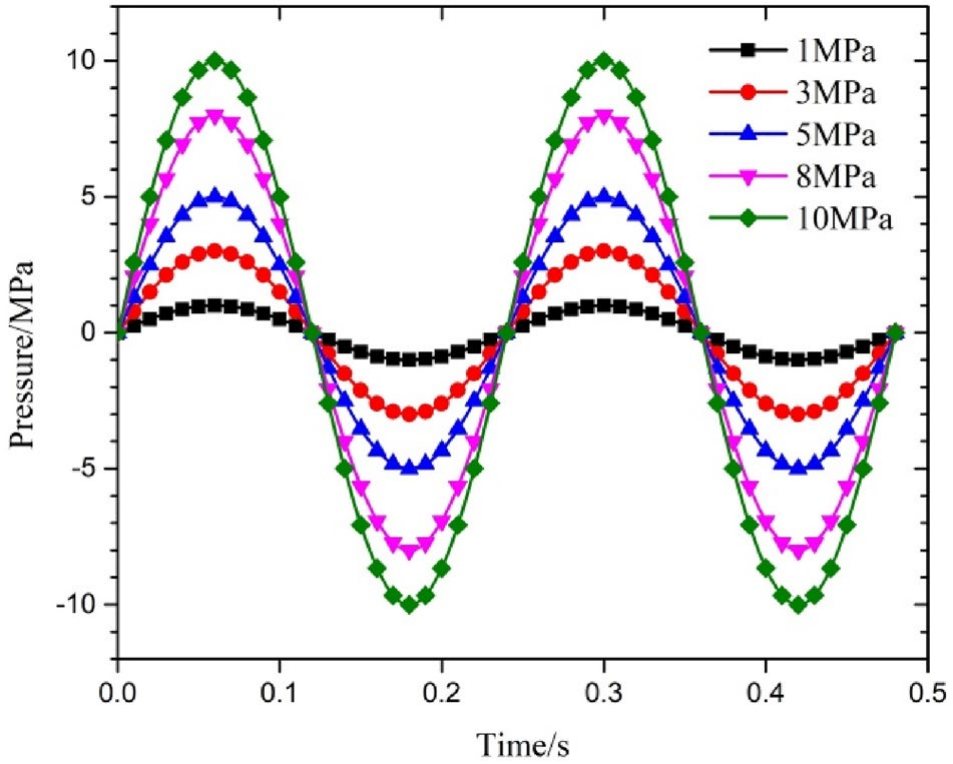
Transient internal pressure with different amplitudes of fluctuation for a period of 0.24 s.

For the loading form of the fluctuation in the internal pressure, based on the study of fluctuation in the axial force, sinusoidal pressure loading was selected, and the fluctuation in the internal pressure was added to the original static pressure. The loading cycle based on the measured minimum cycle time was 0.24 s during the testing; different times included 0.12 s, 0.24 s, 0.36 s, 0.48 s and 0.6 s. The changes in pressure were selected according to the process of switching the well and the measured change in the output; these included 1 MPa, 3 MPa, 5 MPa, 8 MPa and 10 MPa. In order to study the influence of water depth in detail, on the basis of the initial test depth of 660 m, the range of water depth was expanded to 3000 m. When the internal pressure fluctuated, the calculation step was 240 during the two-cycle period. When the period was 0.24 s, the transient load of different amplitudes of fluctuation in the pressure is shown in Fig. 5.

### Equation of transient dynamic analysis

When the FE software package Workbench is used for transient analysis, the solution is based on the equation of transient dynamics:

9 $$\left[ {\mathbf{M}} \right]\left\{ {{\mathbf{\ddot{u}}}} \right\} + \left[ {\mathbf{C}} \right]\left\{ {{\dot{\mathbf{u}}}} \right\} + \left[ {\mathbf{K}} \right]\left\{ {\mathbf{u}} \right\} = {\mathbf{F}}\left( t \right)$$

where $\left[ {\mathbf{M}} \right]$ is the overall mass matrix of the structure, $\left[ {\mathbf{C}} \right]$ is the overall damping matrix of the structure, $\left[ {\mathbf{K}} \right]$ is the overall stiffness matrix of the structure, ${\mathbf{F}}\left( t \right)$ is the external load, and the load form can be any load that changes with time.

The solution of the dynamic response can be used to calculate the deformation, velocity, acceleration, stress and strain of the structure according to variations in the time. The maximum deformation and maximum stress of the string were selected as the response parameters.

## Analysis of the dynamic response of the test string under fluctuations in the axial force

### Analysis of the dynamic response under different amplitudes of fluctuation

**Figure 6:**
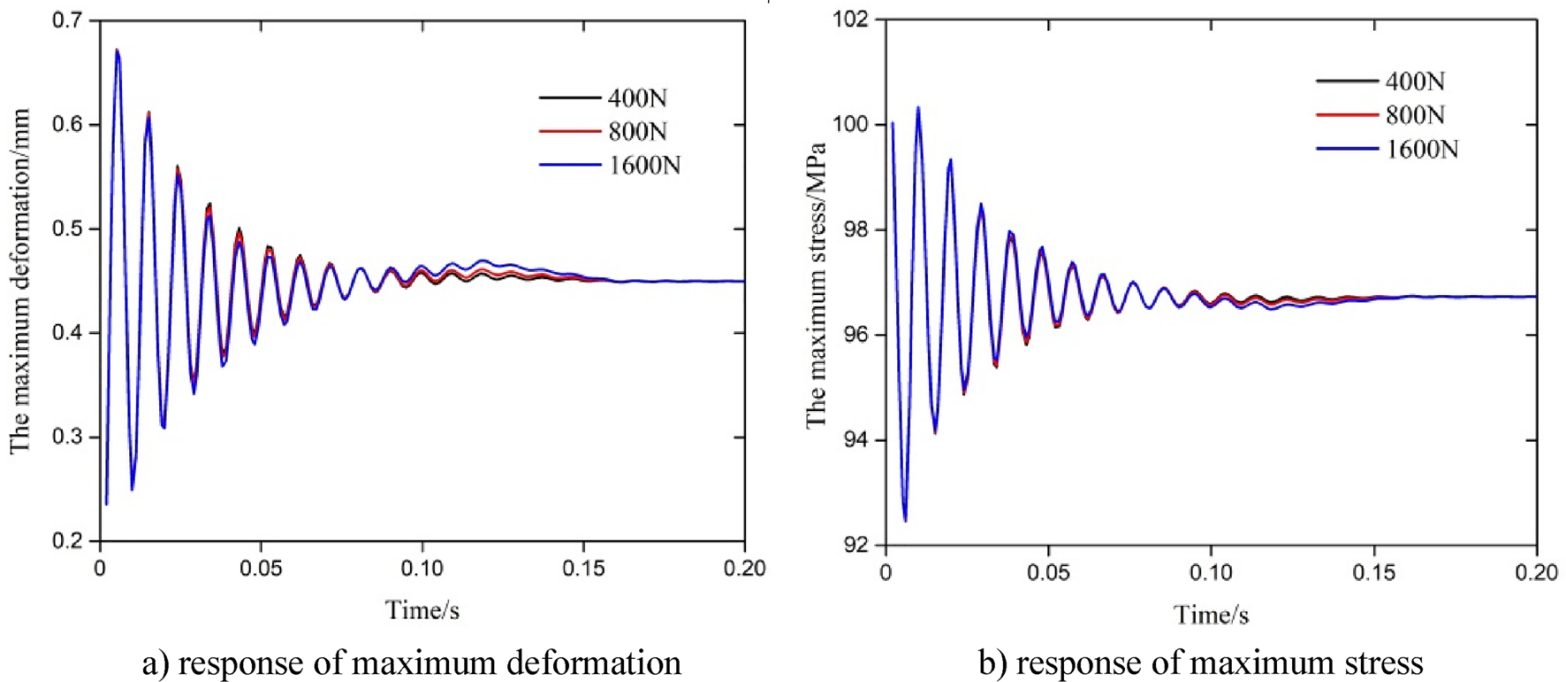
Parametric response under a change in the amplitude of triangular fluctuation in the axial force.

In order to obtain the influence of the fluctuation in the axial force on the dynamic response of the string, the amplitude of fluctuation in the axial force was set to 10% (400 N), 20% (800 N) and 40% (1600 N), and the upper end of string was located at a depth of 475 m. After one cycle, the parameters’ response was almost unchanged under a triangular wave, so the response data for 0–0.2 s was selected for analysis, and the results as shown in Fig. 6.

It can be seen from Fig. 6 that the maximum deformation and maximum stress curves of the string almost completely coincided under the change in the amplitude of fluctuation in the axial force. The parametric response changed obviously in the initial stage of the triangular wave’s action, and tended to stabilize after one cycle (0.16 s). The relevant results show that the dynamic response process of the string was basically identical with a 40% variation in the amplitude of the triangular wave fluctuation in the axial force, and an increase in the amplitude of fluctuation had little effect on the dynamic response of the string in the elastic range. According to Eqs. (4), (6) and (7), the initial triangular fluctuation of axial force causes elastic deformation of the pipe column, and the maximum deformation and stress change with the fluctuation of axial force. Due to friction coupling and Poisson coupling, the small amplitude fluctuation of the axial force of the string gradually weakens, and the maximum stress and displacement tended to stabilize.

### Analysis of the dynamic response of the test string with different water depths

**Figure 7:**
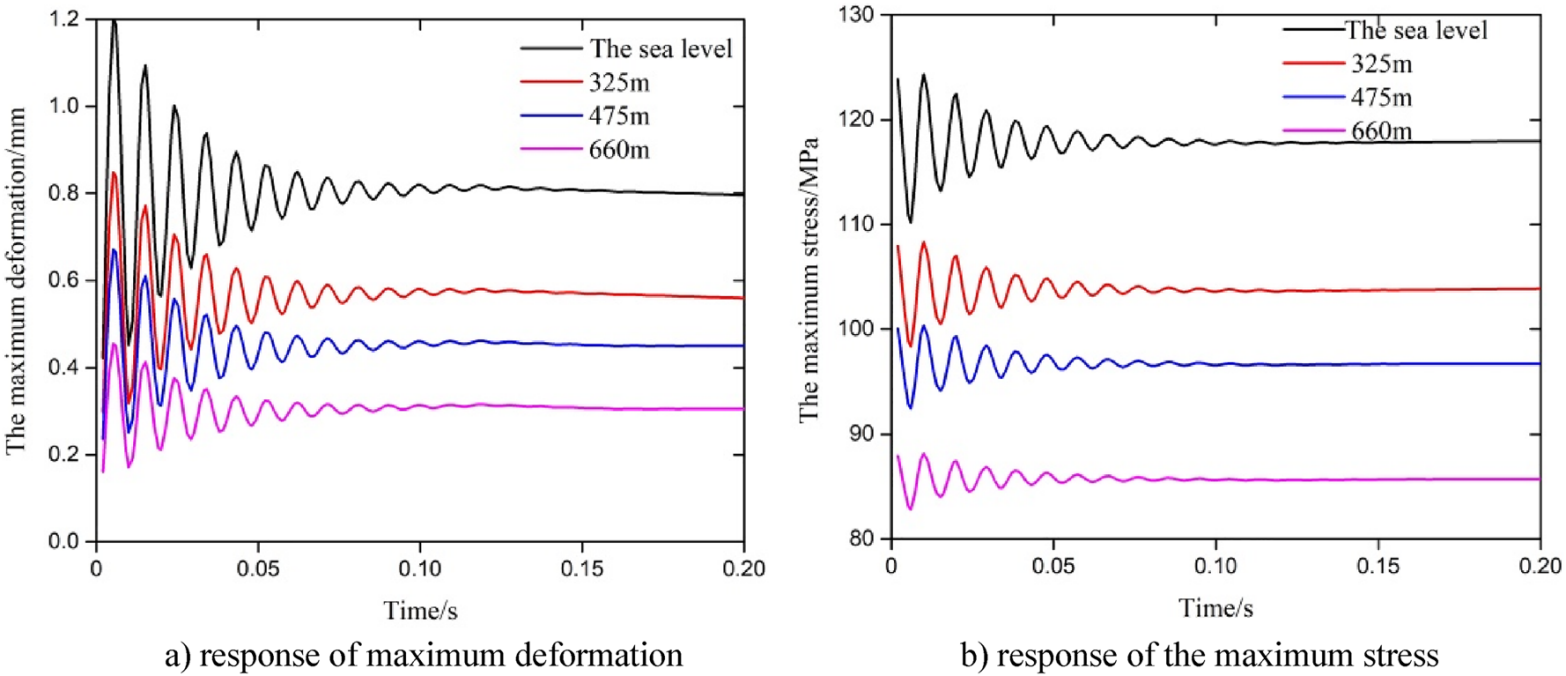
Parametric response under a change of water depth in the triangular fluctuation in the axial force.

The dynamic responses of the test string with different water depths, including the upper end of the string at sea level, 325 m, 475 m and 660 m under triangular fluctuation were analyzed. The results are shown in Fig. 7.

The results in Fig. 7 show that the response law of the maximum deformation and maximum stress of the string at different depths was basically the same. In the early stage of the fluctuation in the axial force, the amplitude of the fluctuation of each response was large, and the fluctuation amplitude gradually decreased with an increase in the time. After one cycle of fluctuation in the axial force, the response tended to become stable. The correlation results also showed that the size of the response parameters decreased with an increase in the water depth. For a single short string (10 m), the maximum value of the maximum stress and displacement during the whole fluctuation are shown in Table 1. The amplitude of the change in maximum deformation under a single wave at the corresponding water depth was 1.049 mm, 0.739 mm, 0.585 mm and 0.396 mm, and the amplitude of the change in maximum stress was 124.33 MPa, 108.29 MPa, 100.32 MPa and 88.129 MPa. It can be seen from the results above that the effect of the internal and external pressure helped to reduce the size of the response parameters of the string with an increase in the water depth. According to Eqs. (4), (6) and (7), the internal and external pressure of the string increased with the increase in water depth, which resulting in the increase of radial stress and hoop stress of the string. While the axial stress remains stable at the initial fluctuation stage, the pressure remains unchanged, and the maximum equivalent stress and displacement fluctuate significantly with the fluctuation of axial stress. As time goes on, the effect of friction coupling and Poisson coupling were significant, and the pressure gradually matches the fluctuation of axial force, radial stress and hoop stress gradually change with axial stress. The changes in axial stress, radial stress, and hoop stress tended to stabilize, therefore, the maximum equivalent stress and displacement tend to stabilize with a certain extent.

**Table 1:** Maximum stress and maximum deformation across time.

Water depths	Maximum stress (MPa)	Maximum deformation (mm)
0 (sea level)	124.33	0.485
325 m	108.29	0.343
475 m	100.32	0.272
660 m	88.129	0.184

### Analysis of the dynamic response of the test string under different frequencies

**Figure 8:**
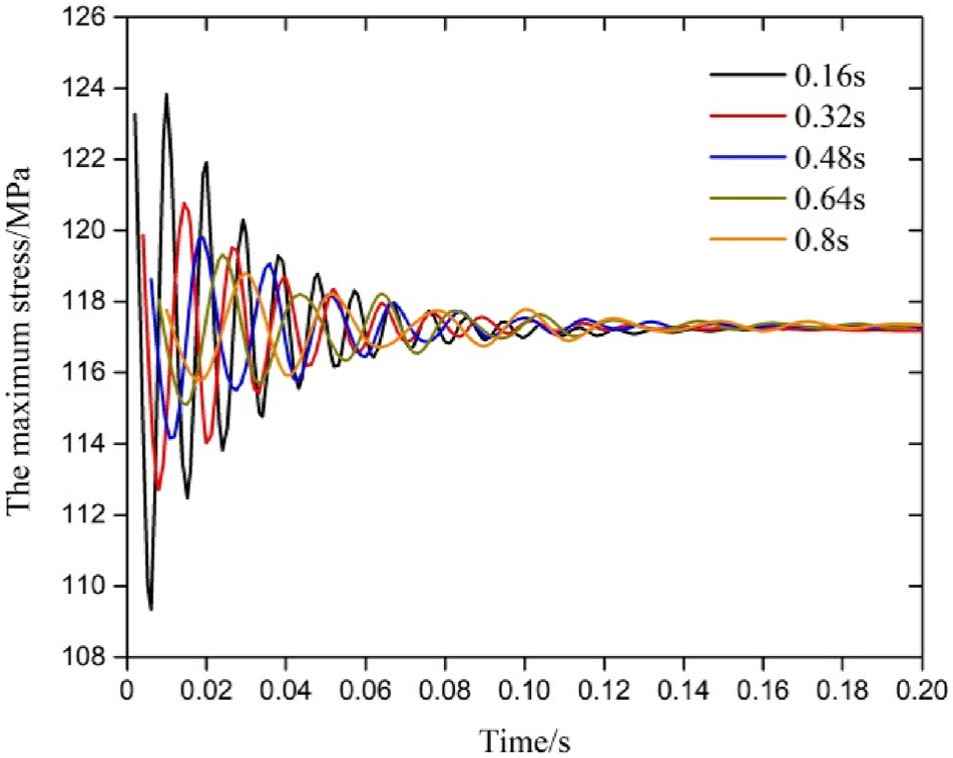
Response of maximum stress across time.

**Figure 9:**
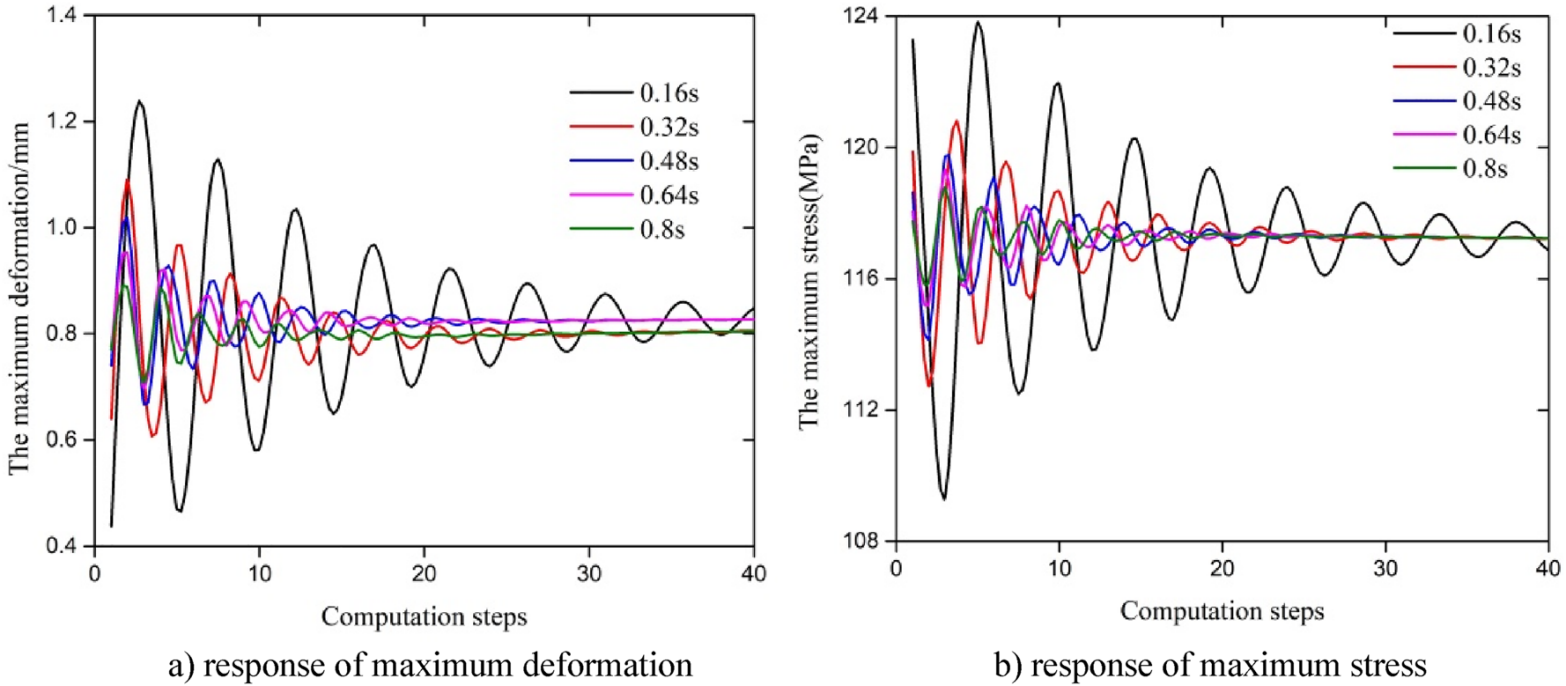
Parametric response under a change in the amplitude of triangular fluctuation of the fluctuation period in the axial force.

According to the results of the analysis, the rate of change in the response parameters decreased when the fluctuation period increased. The results for the first 0.2 s are shown in Fig. 8. Since the variation in the abscissa was very large with different periodic fluctuations, we took the calculation step as the abscissa to compare the variation law of the amplitude of fluctuation with the fluctuation period. The data for the first 40 calculation steps (pre-T/3) were extracted as shown in Fig. 9, and the maximum values of the maximum stress and displacement during the whole fluctuation are shown in Table 2. From Fig. 8 and the frequency of change in the axial force, it can be seen that the change in the response parameters coincided with frequency of fluctuation in the axial force; the change in the amplitude of maximum deformation under a single wave of the corresponding frequency was 1.217 mm, 1.091 mm, 1.014 mm, 0.948 mm and 0.887 mm; and the amplitude of the change in maximum stress under a single wave of the corresponding frequency was 123.27 MPa, 120.11 MPa, 119.73 MPa, 119.32 MPa and118.79 MPa. At the same time, the results in Fig. 9 and Table 2 show that the frequency of fluctuation not only affected the variation in the frequency of the response parameter but also had a great influence on the change in the amplitude of the response with different periods. The amplitude of fluctuation in the response parameters and the fluctuation time ratio (fluctuation time/period duration) increased with an increase in the frequency of the triangular fluctuation in the axial force. According to Eqs. (4), (6) and (7), the increase of axial force fluctuation frequency accelerates the fluctuation of axial stress in the early stage of the string, which leads to friction coupling and Poisson coupling effect to the string. Therefore, the higher the frequency is, the greater the fluctuation amplitude of the maximum displacement and equivalent stress in the initial stage of the string. Overall, due to the unchanged amplitude of axial force fluctuations at different frequencies, the maximum stress and displacement of the string tended to stabilize in the later stage.

**Table 2:** Maximum stress and maximum deformation across time.

Fluctuation period (s)	Maximum stress (MPa)	Maximum deformation (mm)
0.16	123.27	1.217
0.32	120.11	1.091
0.48	119.73	1.014
0.64	119.32	0.948
0.80	118.79	0.887

From Figs. 6, 7, 8 and 9, it also can conclude that the maximum deformation and maximum stress of the string fluctuated significantly in the first period under triangular fluctuation in the axial force with, and the form of the parametric response to fluctuation was a decrease in the amplitude of vibration (damping). The response of the parameters tended to become stable after one period, and the amplitude of fluctuation had little effect on the response parameters. The amplitude of the response parameters decreased with an increase in the water depth. With an increase in the frequency of fluctuation (a decrease in the period of fluctuation), the response frequency of the related parameters increased; the amplitude of the response and the length of time before the fluctuation stabilized increased obviously. The maximum deformation and maximum stress under sine waveform fluctuation in the axial force of the string were smaller than those of a triangular wave, but the stress and strain were all sinusoidal, and with the increase in the amplitude of the axial force, the response of the maximum stress and deformation to changes in the amplitude of fluctuation increased.

### Analysis of the dynamic response of the test string under a change in the amplitude of fluctuation in the axial force with a sine waveform

**Figure 10:**
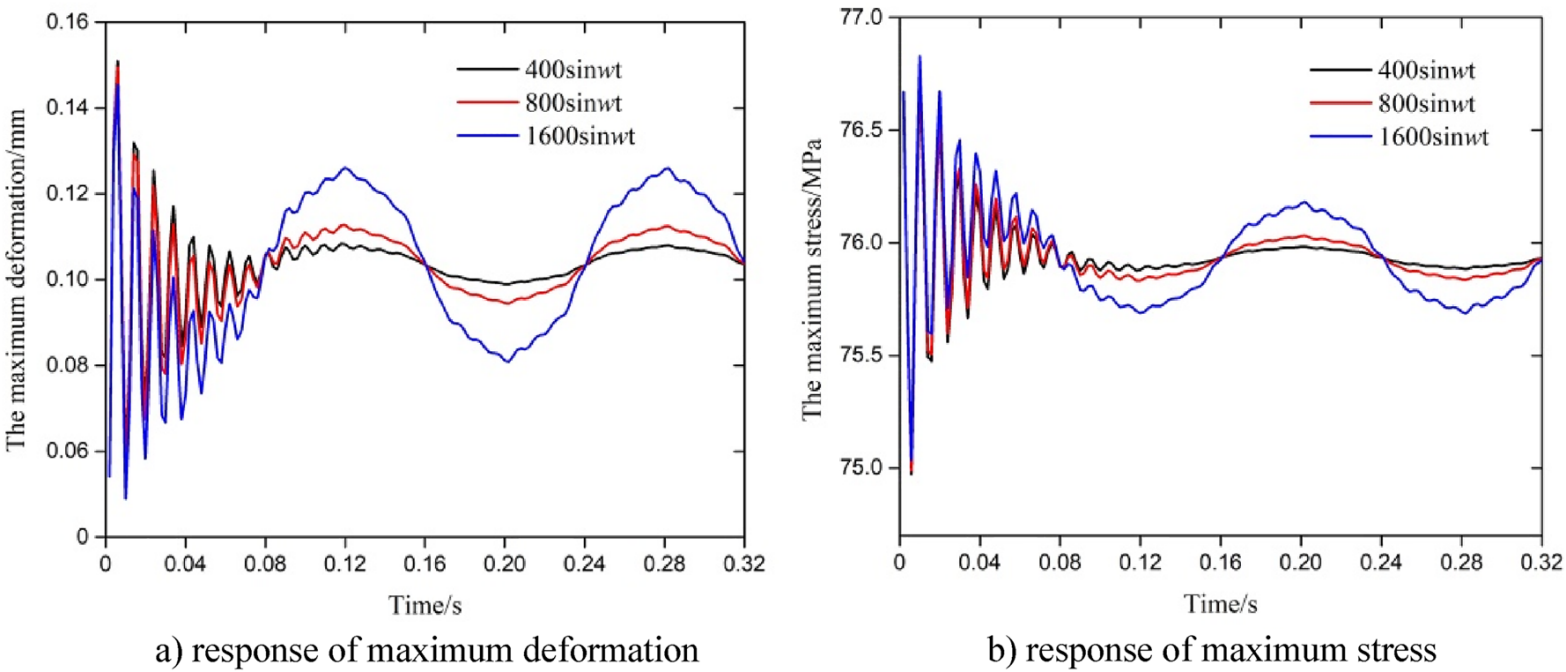
Parametric response under a change in the amplitude of fluctuation in the axial force with sine waveform.

The results in Fig. 10 show that with an increase in the amplitude of fluctuation, the variation in the amplitude of the response parameters increased, and the response parameters changed the damping fluctuation of the maximum deformation and stress in addition to the overall sinusoidal variation, and the local variation was larger in the initial stage of the action. The amplitude of the change in maximum stress and deformation under a single wave showed very small differences. After the axial force changed for nearly half a period, the change in the local response of fluctuation decreased, and the overall fluctuation showed a sine waveform. Compared with the triangular fluctuation of the same amplitude, although the maximum deformation and maximum stress value were smaller than that for the triangular wave, the maximum stress and deformation of the whole showed a sinusoidal change. According to Eqs. (4), (6) and (7), the amplitude of fluctuation in the axial force with a sine waveform resulting in sinusoidal fluctuations in stress. In the initial stage, the axial stress increases with the increase of the amplitude of axial force fluctuation, which resulting in significant changes in the maximum equivalent stress and displacement. Due to friction coupling and Poisson coupling, the fluctuation of axial stress will cause attention to the fluctuation velocity and internal pressure changes of the string, thus resulting in the radial stress and hoop stress changes gradually consistent with the axial stress fluctuation form. Final, the maximum displacement and maximum stress of the string ultimately exhibit a sinusoidal pattern as a whole, and the local fluctuation amplitude is small.

## Analysis of the dynamic responses of the test string under fluctuation in the internal pressure

An analysis of the transient dynamics was carried out according to changes in the internal pressure. Fluctuations in the internal pressure with different periods, different water depths and different amplitudes of fluctuation were analyzed and summarized. When the internal pressure fluctuated, the change in the response parameters was basically consistent with the period of internal pressure. In order to facilitate a comparison of the parameters’ changes for different periods, 240 calculation steps were extracted and used as the abscissa, and the time for one calculation step was T/120.

### The influence of the fluctuation period

**Figure 11:**
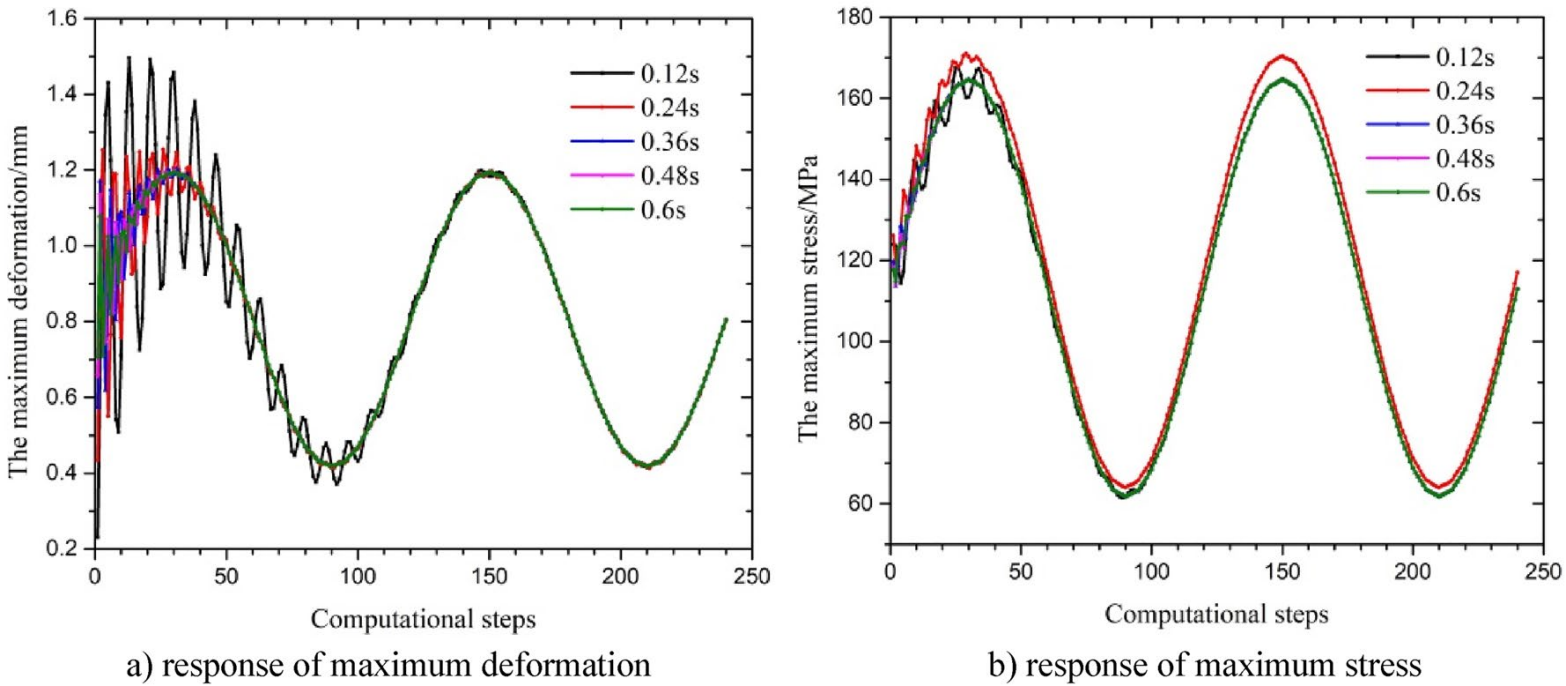
Parametric response under a change in the amplitude of fluctuation period in the internal pressure with a sine wave.

Under different periods of the fluctuation in internal pressure T (0.12 s, 0.24 s, 0.36 s, 0.48 s and 0.6 s), the calculation step was used as the abscissa (the time of the calculation step was T/120), and the results are shown in Fig. 11 and Table 3. The amplitude of the change in maximum deformation under a single wave with the corresponding fluctuation periods was 0.985 mm, 0.818 mm, 0.596 mm, 0.487 mm and 0.372 mm, and the amplitude of the change in maximum stress was very small. The results showed that the influence of the change in the period of internal pressure and the period of the fluctuation in the axial force on the response parameters was basically the same. During the first period of fluctuation in the internal pressure, the amplitude of the response parameters changed greatly and then tended to become stable, and the overall response parameters had a sine waveform. The amplitude of the response and the frequency of fluctuation in the maximum deformation increased with a decrease in the cycle of fluctuation in the internal pressure during the first period, and the fluctuation cycle had a significant influence on the response parameters. The amplitude of the change in maximum deformation reduced by two-thirds. According to Eqs. (4), (6) and (7), the increase of internal pressure fluctuation frequency accelerates the fluctuation of radial stress and hoop stress in the early stage of the string, which also leads to friction coupling and Poisson coupling to the string. Therefore, the higher the frequency is, the greater the fluctuation amplitude of the maximum equivalent stress and displacement in the initial stage of the string. Overall, due to the unchanged amplitude of internal pressure fluctuations at different frequencies, the maximum stress and displacement of the string tended to stabilize in the later stage.

**Table 3:** Maximum stress and maximum deformation across time.

Fluctuation period (s)	Maximum stress (MPa)	Maximum deformation (mm)
0.12	167.56	1.494
0.24	165.47	1.248
0.36	164.74	1.201
0.48	164.56	1.199
0.60	164.62	1.195

### Influence of water depth

**Figure 12:**
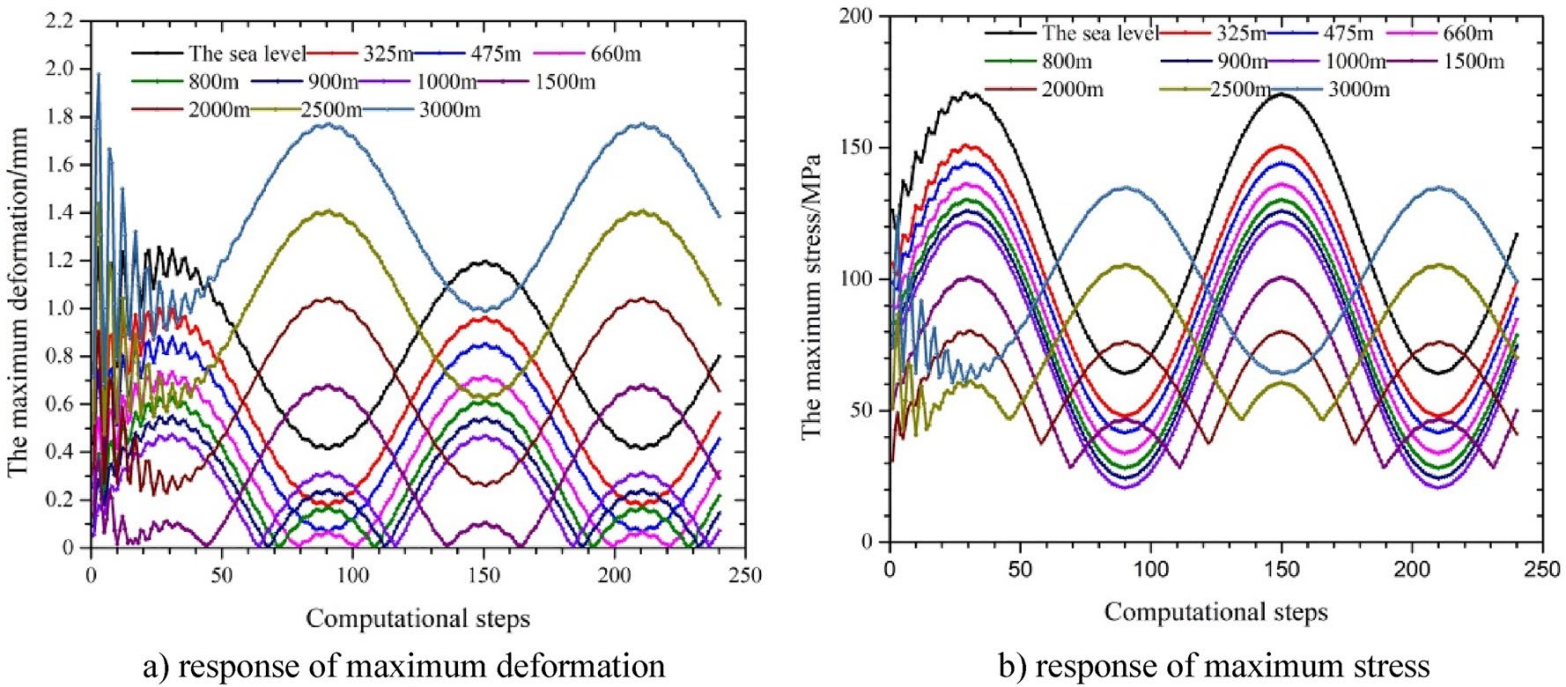
Parametric response under changes in the water depth with sine wave fluctuation in the internal pressure.

In line with the fluctuations in the pressure during field testing operations, the influence of different water depths on the response of maximum stress and deformation were selected when the fluctuation period was 0.24 s, and the results are shown in Fig. 12 and Table 4. The amplitude of the change in maximum deformation under a single wave at the corresponding water depths was 0.818 mm, 0.594 mm, 0.490 mm, 0.361 mm, 0.264 mm, 0.194 mm, 0.115 mm, 0.221 mm, 0.569 mm, 0.969 mm and 1.263 mm. The amplitude of the change in maximum stress was small for depths of 0–1500 m, but when the water depth increased to more than 2000 m, the initial amplitude of fluctuation in stress increased. The results show that the maximum deformation decreased with an increase in the water depth between 0 and 660 m. The amplitude of the change in maximum deformation varied greatly in the first half of period, and then an overall sine waveform trend appeared. The maximum deformation of the string periodically approached zero at depths between 660 and 1500 m in some computational steps; these were mainly a result of the Poisson coupling caused by the change in the internal and external pressure at certain times, which made the equivalent deformation of the string smaller, and the maximum deformation between the minimum two points increased with an increase in the water depth. The maximum deformation fluctuated greatly in the first half-period of fluctuation in the internal pressure when the water depth was 1500 m to 3000 m, then the whole showed a sine waveform. The maximum deformation increased with an increase in the water depth, and the difference in the phase between deformation and changes in internal pressure was close to 90°.

**Table 4:** Maximum stress and maximum deformation across time.

Water depth (m)	Maximum stress (MPa)	Maximum deformation (mm)
0 (sea level)	171.05	1.254
325	150.16	0.999
475	144.39	0.881
660	136.33	0.735
800	130.25	0.624
900	125.91	0.546
1000	121.75	0.467
1500	100.8	0.668
2000	80.362	1.041
2500	105.48	1.407

The results in Fig. 12b show that the maximum stress had a large local fluctuation in the first half of the period and then stabilized. The fluctuation in the maximum stress was in the form of sinusoidal fluctuation between 0 and 1000 m, and the maximum stress value decreased with an increase in the water depth at each calculation step. The response of the maximum stress first increased and then decreased between the two minimum points when the water depth was between 1500 and 3000 m, and the maximum stress of the string increased with an increase in the water depth at arbitrary calculation step. According to Eqs. (4), (6) and (7), the internal and external pressure of the string increased with an increase in the water depth, which resulted in the increase of radial stress and hoop stress of the string. While the axial stress remains constant and the water depth varies greatly, the main stress gradually transitions from axial stress to radial and hoop stress. As a result, the overall trend was that, the maximum equivalent stress decreases first and then increases. Contrary to the change in maximum stress, the increase in internal and external pressure caused by water depth suppresses the change in displacement.

### Influence of the amplitude of fluctuation

**Figure 13:**
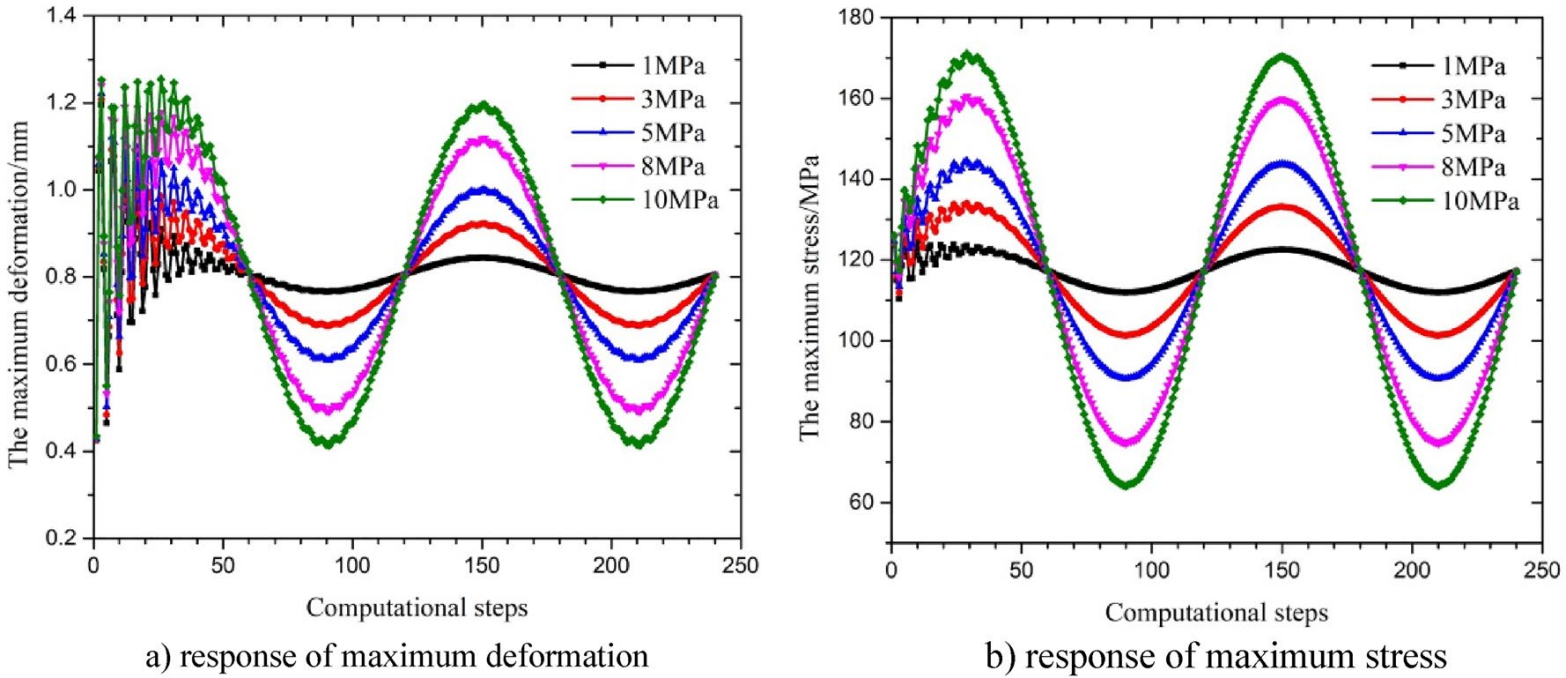
Parametric response under a change in the amplitude of fluctuation in the internal pressure with a sine waveform.

When the internal pressure fluctuates, the amplitude of the change in the pressure is also the main parameter that affects the dynamic response of the string. For this reason, based on the amplitude range of variation in the pressure during the testing operation, the responses of maximum stress and deformation under different amplitudes of fluctuation in the single short string at sea level were analyzed. The results are shown in Fig. 13 and Table 5. The amplitude of the change in maximum deformation under a single wave with the corresponding fluctuation periods was 0.770 mm, 0.781 mm, 0.792 mm, 0.808 mm and 0.819 mm, and the amplitude of the change in maximum stress was very small. The results show that with an increase in the amplitude of fluctuation, the amplitude of fluctuation in the maximum stress and deformation increased. The maximum stress and deformation changed obviously in the first half of the period of fluctuation in the pressure, and then the maximum stress and maximum deformation were similar to the sine waveform of the fluctuation in the internal pressure. According to Eqs. (4), (6) and (7), the increase of internal pressure fluctuation amplitude accelerates the fluctuation of radial stress and hoop stress, which also leads to friction coupling and Poisson coupling to the string in the initial stage of the string. Therefore, the larger of the pressure amplitude, the increase in radial stress and hoop stress in string leads to significant changes at maximum displacement and equivalent stress in the initial stage. Overall, the maximum stress and displacement with small fluctuationsin the later stage.

**Table 5:** Maximum stress and maximum deformation across time.

Fluctuation amplitude (MPa)	Maximum stress (MPa)	Maximum deformation (mm)
1	125.01	1.195
3	123.99	1.208
5	144.58	1.221
8	160.46	1.240
10	171.05	1.254

## Conclusion

Through the comprehensive consideration of the string's floating weight coefficient, faux force, tension, frictional resistance, the force of the variable diameter, etc., a method of calculating the axial force applicable to the seawater section of a test string was derived, and a model of the transient dynamics of the longitudinal vibration and transverse vibration suitable for a deepwater test string above the mud line was established. Through the established FE model of the equal-section string, an analysis of the dynamic response to fluctuation in the axial force and the internal pressure of the test string was carried out by using Workbench. The main conclusions are as follows:

1. The influence of fluctuation in the axial force and internal pressure on the dynamic response of the string occurred mainly in the initial stage of the fluctuation.
2. The dynamic parameters of string tend to stabilize after the first cycle of axial force fluctuations under a change in the amplitude of triangular fluctuation in the axial force, the internal and external pressure helped to reduce the size of the response parameters of the string with an increase in the water depth, and the fluctuation of response parameters increased with the increase of the frequency.
3. The maximum deformation and maximum stress under sine waveform fluctuation in the axial force of the string were smaller than those of a triangular wave, but the stress and strain were all sinusoidal, and with the increase in the amplitude of the axial force, the response of the maximum stress and deformation to changes in the amplitude of fluctuation increased.
4. The amplitude of change in the response parameters increased with an increase in the sinusoidal amplitude of internal pressure. The intense response of maximum stress and maximum deformation occurred in the first half-period of fluctuation, and the fluctuation of response parameters increased with a decrease in the cycle of fluctuation in the internal pressure.
5. The internal and external pressure increased with an increase in water depth increases, which resulted in increase of the radial stress and hoop stress, and led to complex changes in dynamic parameters ultimately.
6. The maximum deformation and stress of the string always changed with the load, and the string under a sine waveform load was more prone to periodic fatigue failure. Future studies of the amplitude of fluctuation under a high load could focus on the processes of opening the test well, shut-in, yield adjustment and so on, as well as subsequent safety analyses of the test string.

## Data Availability

The datasets used and/or analysed during the current study available from the corresponding author on reasonable request.
